# KAPS (kinematic assessment of passive stretch): a tool to assess elbow flexor and extensor spasticity after stroke using a robotic exoskeleton

**DOI:** 10.1186/s12984-017-0272-8

**Published:** 2017-06-19

**Authors:** Andrew Centen, Catherine R. Lowrey, Stephen H. Scott, Ting-Ting Yeh, George Mochizuki

**Affiliations:** 1 Heart and Stroke Foundation Canadian Partnership for Stroke Recovery, Sunnybrook Research Institute, Toronto, ON Canada; 2 Brain Sciences Research Program, Sunnybrook Research Institute, 2075 Bayview Avenue, M6-178, Toronto, ON M4N 3M5 Canada; 3 Department of Physical Therapy, Faculty of Medicine, University of Toronto, Toronto, ON Canada; 4 Toronto Rehabilitation Institute – University Health Network, Toronto, ON Canada; 5 Centre for Neuroscience Studies, Queen’s University, Kingston, ON Canada; 6 Department of Biomedical and Molecular Sciences, Queen’s University, Kingston, ON Canada; 7 Present Address: School of Occupational Therapy, College of Medicine, National Taiwan University, Taipei, Taiwan

**Keywords:** Stroke, Spasticity, Robotics, Upper extremity

## Abstract

**Background:**

Spasticity is a common sequela of stroke. Traditional assessment methods include relatively coarse scales that may not capture all characteristics of elevated muscle tone. Thus, the aim of this study was to develop a tool to quantitatively assess post-stroke spasticity in the upper extremity.

**Methods:**

Ninety-six healthy individuals and 46 individuals with stroke participated in this study. The kinematic assessment of passive stretch (KAPS) protocol consisted of passive elbow stretch in flexion and extension across an 80° range in 5 movement durations. Seven parameters were identified and assessed to characterize spasticity (peak velocity, final angle, creep (or release), between-arm peak velocity difference, between-arm final angle, between-arm creep, and between-arm catch angle).

**Results:**

The fastest movement duration (600 ms) was most effective at identifying impairment in each parameter associated with spasticity. A decrease in peak velocity during passive stretch between the affected and unaffected limb was most effective at identifying individuals as impaired. Spasticity was also associated with a decreased passive range (final angle) and a classic ‘catch and release’ as seen through between-arm catch and creep metrics.

**Conclusions:**

The KAPS protocol and robotic technology can provide a sensitive and quantitative assessment of post-stroke elbow spasticity not currently attainable through traditional measures.

## Background

Potential targets for neuromotor rehabilitation after stroke are guided by information obtained from sensorimotor assessments. The most ideal assessments are sensitive to relevant change and objective as they are used to monitor recovery and may determine the efficacy of an intervention. Careful measurement of sensorimotor impairments is essential for optimizing stroke recovery outcomes.

Spasticity is one neurophysiological consequence of stroke characterized as excessive muscle contraction in response to stretch and reflects velocity-dependent resistance to passive movement [1, 2]. Using clinical assessments, the prevalence of spasticity after stroke is estimated to be between 17 and 27% [3, 4] but has been reported to be as high as 38% [5]. The variability in prevalence estimates may be attributable, in part, to the subjective and relatively coarse methods for measuring resistance to passive movement that is routinely used in the clinical setting.

Most commonly assessed using the Modified Ashworth Scale (MAS) [6] and the Modified Tardieu Scale (MTS) [7], spasticity is measured clinically by passively moving a joint through its range at a speed equivalent to counting “one-thousand-and-one” [6] (i.e. MAS) or at three speeds [7] (i.e. MTS) while grading the extent and angle of muscle contraction. The MAS is a relatively coarse scale with 6 levels (ranging from no resistance to rigid). Inter-rater reliability has been excellent (Kendall’s tau 0.847) [6]; however, more recent evidence suggests only moderate inter-rater reliability (K 0.42) and intra-rater reliability (K 0.53) [8]. The MTS can differentiate spasticity from contracture [9], though only moderate-to-good reliability is achieved (ICC 0.56 to 0.74) [10] and it is difficult to draw conclusions on the validity of the MTS due to a limited amount of literature [11]. The psychometric properties of these assessments indicate that they are moderately suited to assess spasticity and that there is a need for a more objective approach to accurately assess spasticity as a means of determining effects of treatment or guiding rehabilitation strategies.

To overcome the limitations with using the MAS and MTS, attempts have been made to develop instrumented assessments to quantify resistance to passive movement in neurological populations including stroke and cerebral palsy [12, 13]. These assessments have included inertial sensors for improved reliability over hand-held goniometers [14], load cells or force transducers for higher control of stretch [15–17], and electrogoniometers and electromyography for determining dynamic and tonic stretch reflex thresholds [18, 19]. These techniques may overcome some limitations associated with the MAS and MTS but, in spite of the enhanced control of movement, they still require manual stretch and/or use of EMG signals which require additional expertise and time for interpretation. Advanced technologies like position controlled servomotors [20] and torque motors [21, 22] have provided opportunities to objectively probe spasticity. Katz and Rymer developed a servo-controlled robotic device capable of applying ramp and hold movements to the upper extremity that quantified the angle at which reflex torque and EMG begin to increase in response to stretch [23]. However, the authors suggested that for instrumented measurement of spastic hypertonia to be clinically meaningful, it must be referenced to a set of normative comparators. In so doing, the vast heterogeneity in clinical characteristics of movement kinematics and in the etiologies of increased muscle tone (i.e. both neurogenic and non-neurogenic factors) can be normalized to idealized movement patterns to determine the impact of increased muscle tone on passive movement kinematics. Recent work by Seth and colleagues [24] has addressed this requirement and has identified differences in measures between healthy controls and individuals with stroke with MAS = 0.

The objective of this study was to develop a tool to assess post-stroke spasticity in the upper limb. An assessment using a robotic exoskeleton was designed to: A) identify unique kinematic characteristics associated with resistance to passive movement in comparison to a neurologically intact cohort and; B) provide a standardized methodology for assessment of both elbow flexors and elbow extensors. Through full weight support of the upper limb with tight control over the velocity and range of passive movement, this tool aimed to provide objective and sensitive metrics of the extent, onset, and persistence of resistance to passive movement of both the elbow flexors and extensors in post-stroke individuals with and without clinically identified spasticity. The inclusion of a non-spastic stroke group was meant to determine whether the tool possessed the sensitivity to detect features characterizing resistance to movement that were not identified using traditional clinical measures.

## Methods

### Participants

Participants were recruited from the inpatient stroke unit and outpatient spasticity clinic at the Toronto Rehabilitation Institute and outpatient spasticity services at Sunnybrook Health Sciences Centre in Toronto, Canada. Participants with stroke were 18 years of age or older, at least 2 weeks post ischemic or hemorrhagic stroke, able to maintain 90° shoulder abduction without pain, had normal or corrected-to-normal vision, and were able to understand instructions and provide written consent. Individuals were excluded if they had a communication limitation that would prevent expressing concern or desire to withdraw, a pre-existing neurological condition, or cognitive/behavioural issues that could influence testing.

Healthy individuals were recruited to create a control database if they satisfied the applicable criteria outlined above. Control participants were recruited through a convenience sample from the Toronto Rehabilitation Institute and from the community in Kingston, Canada. All participants provided written informed consent. Procedures were approved by Toronto Rehabilitation Institute, University of Toronto, and Queen’s University ethics boards.

### Experimental setup

**Fig. 1:**
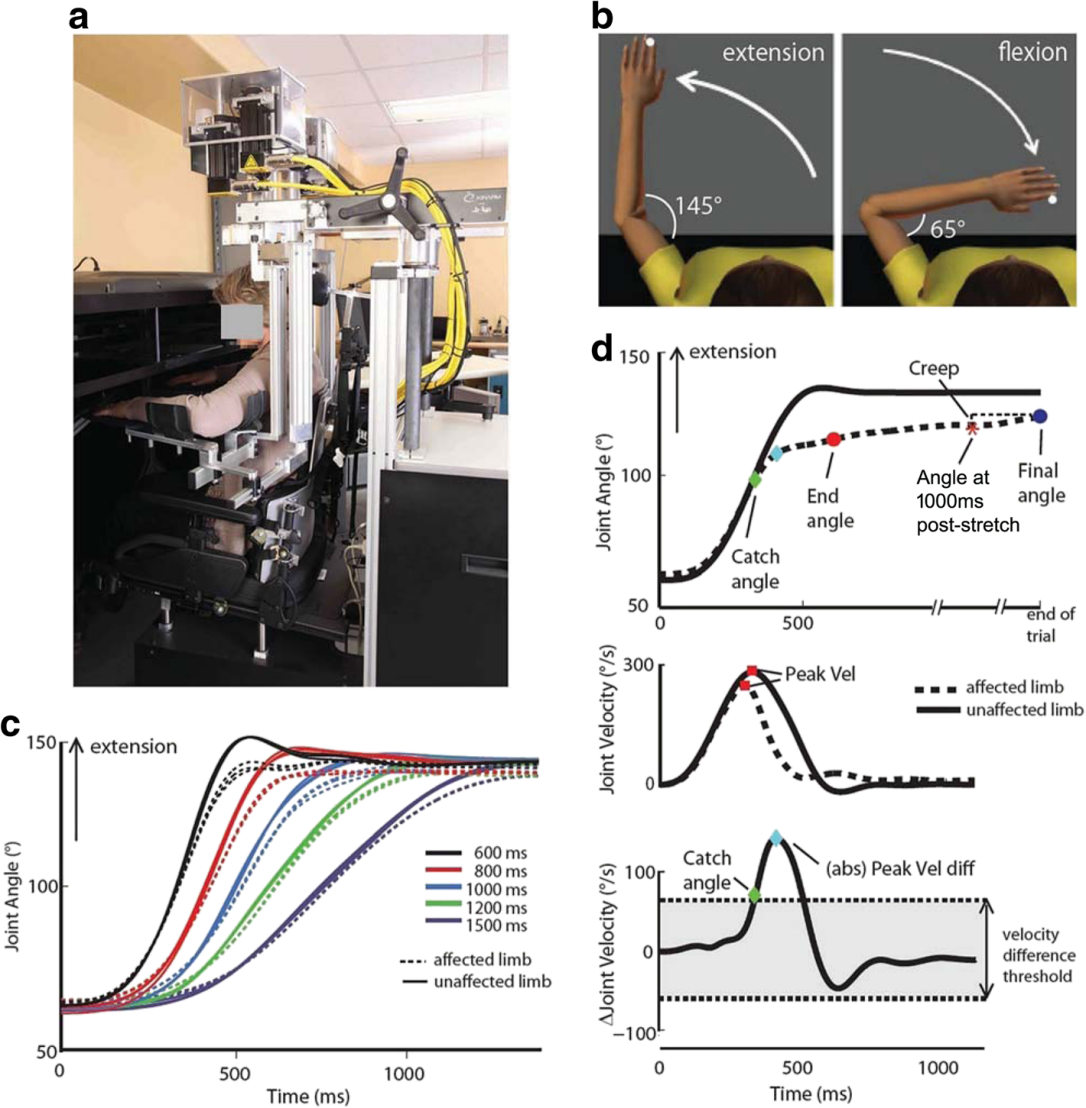
**a** Diagram of a participant seated at the KINARM robotic exoskeleton. **b** Schematic of Kinematic Assessment of Passive Stretch (KAPS) protocol. **c** Angular trajectory for an exemplar participant with stroke illustrating the consistency across the three trials for each movement duration (600, 800, 1000, 1200 and 1500 ms). **d** Parameter calculations (represented for 600 ms trial). *Top trace*: Catch angle (*green diamond*) is calculated as the first point in time where the velocity of the affected limb (*dotted line*) deviates from the nominal velocity from the unaffected limb (*solid line*). End angle is the angular position at the end of the movement. Final angle (*blue circle*) is the angular position at the end of the trial (2500–6000 after movement end). Creep was calculated as the difference between the elbow angle at 1000 ms after nominal movement duration (*red star*, Angle at 1000 ms post-stretch) and the Final angle. *Middle trace*: Peak velocity (*red square*) was calculated for both arms. Bottom trace: Peak velocity difference (*blue diamond*) was calculated as the peak of the absolute difference in velocity between the limbs. Velocity difference threshold (*bottom trace, gray shaded area*) was calculated based on the 99th percentile of healthy control performance. A catch angle was recorded only if the between-arm velocity difference for stroke participants exceeded the control velocity difference threshold

An automated approach to assessing elbow spasticity was developed using the KINARM robotic exoskeleton for the upper limb (BKIN Technologies Ltd., Kingston, Canada) [25]. KINARM is capable of applying loads at either the shoulder or elbow joints using torque motors while angular position and angular velocity are recorded. A detailed description has been presented previously [26, 27]. Briefly, participants were seated with arms resting in troughs with shoulders abducted ~85° (Fig. 1a). Shoulder and elbow joints were aligned with the linkages of the robot and movement was permitted in the horizontal plane. Physical stops did not allow the elbow to hyperextend and in instances of passive stretch torque was limited to 13.2 Nm. Elbow angle, fingertip position, and segment length for both arms were calibrated for each participant.

### Assessment protocol

The kinematic assessment of passive stretch (KAPS) protocol designed for this study consisted of passive elbow flexion and extension movements between 65and 145° (internal elbow angle; Fig. 1b) with the shoulder flexed at a fixed 60° (relative to coronal plane). A trial consisted of a flexion movement from 145° towards 65°, followed by a 1500 ms wait, and an extension movement back to 145°. At the end of each trial there was a 3000 ms wait to minimize effects of repeated stretching on subsequent responses while keeping the total time to complete the task low. Each movement was programmed to occur over a specific duration where the 80° range was covered in either 1500 ms (slowest stretch), 1200 ms, 1000 ms, 800 ms, or 600 ms (fastest stretch). The 1000 ms speed was initially chosen to parallel the timing employed when administering the MAS. The other steps in the range were arbitrarily chosen, with the exception of the fastest stretch (600 ms) which was used with patient safety (guarding against joint injury) in mind. A total of 3 trials at each duration were completed; all trials for the 1500 ms condition were completed first, followed by all trials for the 1200 ms condition, and so forth until 15 trials were complete (15 flexion movements and 15 extension movements). The durations of movement were not randomized as there was negligible progressive change across trials during pilot testing. Joint angles for 15 extension movements of the affected and less-affected side of an exemplar participant are illustrated in Fig. 1c. The more affected side was assessed first followed by the less affected side.

The position controller of the robot used joint kinematics, robot inertias, and the position control set point to calculate the motor torques required to reach the target angle in the specified duration. A trajectory was defined based on a sine function that specified elbow joint angle scaled to the duration of the stretch with a feedforward model included to account for robot inertia and an estimate of subject inertia. The proportional gain was 30 Nm/rad and the derivative was 2.5 Nm/(rad/s). The gains were subjectively selected to be high enough to provide relatively similar joint motions across healthy subjects, but low enough to generate measureable deviations in joint motion if a subject generated any resistance. In instances where the target angle was not reached due to participant resistance, the position controller of the robot continued to produce torques, within safe bounds, for up to 7000 ms after movement onset in attempt to reach the target angle. If the target angle was reached within 7000 ms after movement onset, the task continued as described above. If the target angle was not reached, the current position at 7000 ms after movement onset became the new start angle for the next movement.

### Outcome measures

Elbow angular position and velocity were sampled at 1000 Hz using Dexterit-E (BKIN Technologies Ltd., Kingston, Canada). A Butterworth low-pass filter with a cut-off frequency of 10 Hz was applied using Matlab (The MathWorks Inc., Natick, MA, USA). Three parameters were calculated for each arm individually, and an additional 4 parameters were calculated as differences between the more affected and less affected arm (stroke) or non-dominant and dominant arms (control) for a total of 7 parameters (Fig. 1d). Each parameter was calculated for flexion and extension movements separately, and included:

- Peak velocity – maximum angular elbow velocity.
- Final angle – elbow angle at the final recorded position before start of next movement.
- Creep – difference between the elbow angle at 1000 ms after nominal movement duration (Angle at 1000 ms post-stretch) and the Final angle. Positive Creep always reflected further movement toward the Final angle in the direction of the imposed movement. Thus it was calculated differently for flexion and extension movements:
  Flexion Creep = (Angle at 1000 ms post-stretch) – Final angle
  Extension Creep = Final angle – (Angle at 1000 ms post-stretch)
- Between-arm peak velocity difference – absolute difference in Peak velocity between arms.
- Between-arm final angle difference – difference in the Final angle between arms
  (more affected/non-dominant – less affected/dominant).
- Between-arm creep difference – difference in Creep between the arms
  (more affected/non-dominant – less affected/dominant).
- Between-arm catch angle – the angle at which the Between-arm peak velocity difference exceeded the velocity difference threshold (positive value for extension, negative value for flexion). The velocity difference threshold was set at 99% of Between-arm peak velocity difference calculated from the control participants (threshold = 50.1°/s). If the difference in velocity did not exceed the threshold, no catch angle was recorded.

The 3 single limb measures (peak velocity, final angle, and creep) were developed to capture features that are considered when clinical scoring is conducted using the MAS. Peak velocity represented the maximum angular elbow velocity during movement. Final angle was used to characterize passive range of motion. Creep was used to characterize ‘release’ after the ‘catch’. Thus, one would expect that more severe spasticity would be characterized by lower velocity due to increased resistance to passive movement, a reduced final angle due to a shortened range of motion, and increased creep because of increased stiffness part way through the range of motion. The between-limb measures were used to enable within-person normalization of data to identify the extent of (dis)similarity between the affected and less affected (or dominant/non-dominant) limbs in peak velocity, creep, final angle, and catch angle.

### Clinical assessment

Spasticity was measured clinically using the MAS and MTS, administered by one of two physiotherapists or a trained study investigator (AC or TTY). Elbow flexor and extensor spasticity were assessed on the more affected side 30 min prior to the KINARM assessment whenever possible to minimize time-of-day effects that reportedly lead to variability of the clinical evaluation of spasticity and the behaviour of the stretch reflex [21, 28]. By minimizing the time between clinical assessment and KINARM assessment, the severity of spasticity should be unchanged. The MAS and MTS were administered with the participant sitting comfortably, otherwise standardized according to traditional use of these scales [6, 7]. The Chedoke McMaster Stroke Assessment (Arm Score) was used to characterize the extent of motor impairment [29]. For participants recruited through inpatient services, score at admission was used. For participants recruited through outpatient services, a trained physiotherapist performed the assessment within 2-weeks of the KINARM assessment. Score at admission and scores within 2-weeks of the KINARM assessment were not always available; these are listed as ‘unknown’ in Table 1.

**Table 1:** Patient Information & Clinical Scores

Measure		Group				
		No Spasticity *n* = 17	Flexors Only *n* = 13	Extensors Only *n* = 3	Both Muscle Groups *n* = 13	Healthy *n* = 96
Age^a^		59 (22, 92)	63 (18, 80)	52 (45, 79)	56 (27, 75)	40 (19, 81)
Sex (M:F)		11:6	11:2	2:1	8:5	41:55
Handedness (R:L)		16:1	12:1	3:0	11:2	88:8
Patient Type (Out:In)		6:11	10:3	3:0	10:3	-
Days Since Stroke^a^		41 (14, 1313)	341 (21, 6195)	542 (280, 662)	728 (14, 4510)	-
Lesion Type (I:H:U)		9:5:3	9:1:3	1:2:0	9:3:1	-
More-Affected Arm (R:L)		10:7	4:9	2:1	8:5	-
CMSAarm Score^b^		[0,1,0,1,2,1,0,12]	[0,2,3,2,2,0,0,4]	[0,3,0,0,0,0,0,0]	[0,4,1,3,1,0,0,4]	-
MTS(V3)^c^	Flexors	[17,0,0,0,0]	[0,0,13,0,0]	[3,0,0,0,0]	[0,1,12,0,0]	-
	Extensors	[17,0,0,0,0]	[13,0,0,0,0]	[0,0,3,0,0]	[0,3,10,0,0]	-
MAS^d^	Flexors	[17,0,0,0,0,0]	[0,1,7,5,0,0]	[2,1,0,0,0,0]	[0,2,9,2,0,0]	-
	Extensors	[17,0,0,0,0,0]	[13,0,0,0,0,0]	[0,1,1,0,1,0]	[0,5,6,2,0,0]	-

*Abbreviations*: *M:F* Male:Female, *R:L* Right:Left, *Out:In* Outpatient:Inpatient, *I:H:U* Ischemic:Hemorrhagic:Unknown, *CMSAarm* Chedoke-McMaster Stroke Assessment – Impairment Scale Arm, *MTS(V3)* Modified Tardieu Scale, fastest speed, *MAS* Modified Ashworth Scale

^a^median value, minimum and maximum values in parentheses

^b^[n_1_, n_2_, n_3_, n_4_, n_5_, n_6_, n_7_, unknown] represents number of participants with CMSA arm scores of [1–7]

^c^[n_1_, n_2_, n_3_, n_4_, n_5_] represents number of participants with MTS scores of [0, 1, 2, 3, 4]

^d^[n_1_, n_2_, n_3_, n_4_, n_5_, n_6_] represents number of participants with MAS scores of [0, 1, 1+, 2, 3, 4]

### Statistical analyses

Statistical analyses were performed using Matlab (The MathWorks Inc., Natick, MA, USA). Full details on statistical analyses have been previously reported [30]. Briefly, control data were age-regressed and then normalized where possible using Box Cox transforms [31]. Control participants identified as outliers in any parameter were removed from all subsequent analyses (*n* = 8). Parameters were then assessed for sex and handedness effects. When parameters were unable to be normalized, non-parametric tests were used. Percentiles were calculated for each parameter and used as cut-off values for comparing individual stroke kinematics. A subject was identified as impaired on a given outcome measure if their performance was <5^th^ or >95^th^ percentile (See Table 2). Failure rate for each measure quantified the percentage of all participants within a participant group (described below) who were impaired based on 5th or 95th percentile cut off values. Z scores were calculated for those parameters that were normal or transformed to normal and were used to reflect degree of failure on a measure. Correlations between parameter and clinical scores were performed using Spearman’s rank correlation.

**Table 2:** Stroke Participant Task Performance

	Cut off percentile	Healthy Control Data *n* = 96	No Spasticity *n* = 17	Flexors Only *n* = 13	Extensors Only *n* = 3	Both Muscle Groups *n* = 13	Inter Rater (ICC)	Clinical Correlations			
								MAS	MTS		
Extension	(%)	Mean (cut off value)	(%)	(%)	(%)	(%)	(r)		V1	V2	V3
Peak Velocity (°/s)	<5	306 (286)	41	77	100	92	0.90	*-0.47*	−0.33	*-0.51*	*-0.51*
Final Angle (°)	<5	M: 143 (140) F: 144 (141)	18	54	67	85	0.86	*-0.46*	−0.34	*-0.44*	−0.39
Creep (°)	>95	−0.05 (0.16)	12	54	67	77	0.66	*0.55*	0.40	*0.44*	*0.45*
*Difference*											
Peak Velocity (°/s)	>95	M: 5.4 (33.5) F: 3.1 (27.5)	47	85	100	92	0.95	0.15	−0.25	0.04	0.09
Final Angle (°)	<5	0.19 (−1.9)	29	77	100	85	0.91	*-0.49*	−0.35	*-0.49*	*-0.45*
Creep (°)	>95	M:0.09 (0.26) F: 0.04 (0.37)	24	62	33	77	0.74	*0.59*	0.40	*0.44*	*0.45*
Flexion											
Peak Velocity (°/s)	>95	M: 269 (240) F: 280 (255)	41	62	100	92	0.87	−0.34	−0.27	−0.38	−0.34
Final Angle (°)	<5	M: 68 (69) F: 67 (70)	47	38	100	77	0.84	*0.46*	0.32	*0.53*	*0.45*
Creep (°)	>95	−0.04 (0.28)	24	85	100	92	0.86	*0.62*	0.34	*0.43*	*0.57*
*Difference*											
Peak Velocity (°/s)	>95	M:-1.5(33.7) F: −0.9 (30.7)	59	85	100	92	0.88	0.12	0.30	0.17	0.17
Final Angle (°)	>95	−0.01 (2.06)	18	46	100	54	0.88	*0.50*	0.32	*0.48*	*0.48*
Creep (°)	>95	0.009 (0.41)	12	62	67	92	0.79	*0.64*	0.36	*0.58*	*0.55*

*Abbreviations*: *M* male, *F* female, *ICC* Intra-class Correlation, *MAS* Modified Ashworth Scale, *MTS* Modified Tardieu Scale, *V1–3 reflect the 3 test speeds* V1 is slow as possible, V2 is speed of limb falling under gravity, V3 is moving as fast as possible

Difference parameters are the between-arm differences. Italicized values for clinical correlations represent *P* < 0.004 (Bonferroni corrected, *n* = 12 tests)

Intra-class correlation coefficients (r-values) were calculated for a sub-sample of participants (19 stroke and 10 control) tested by two separate examiners to test the degree of absolute agreement. The KINARM™ was set up by a trained study investigator or trained operator and the assessment carried out. Upon completion of the assessment, the same operator reset the robot to a random position and a new trained investigator or operator repeated the set up and assessment.

## Results

### Participant information

Demographics and clinical information for 46 individuals with stroke (patient population) and 96 healthy individuals (control population) that participated in this study are presented in Table 1. The patient population was grouped according to MTS score during the fastest stretch based on the absence (No spasticity, *n* = 17) or presence (Spasticity, *n* = 29) of resistance to passive movement. The Spasticity group was further divided according to muscle groups affected: flexors only (*n* = 13), extensors only (*n* = 3), and both muscle groups (*n* = 13). There was agreement between the MAS and MTS for all participants in the no spasticity group and all but 1 participant in the spasticity group. Nineteen individuals with stroke and 10 healthy controls were separately assessed and subsequently included in the intra-class correlation analyses with 8 of 19 included in the main analysis. The remaining 11 individuals who were included in the intra-class correlation were not grouped or included in Table 1 as only the MAS was tested and the MTS was not administered.

### General kinematic pattern

**Fig. 2:**
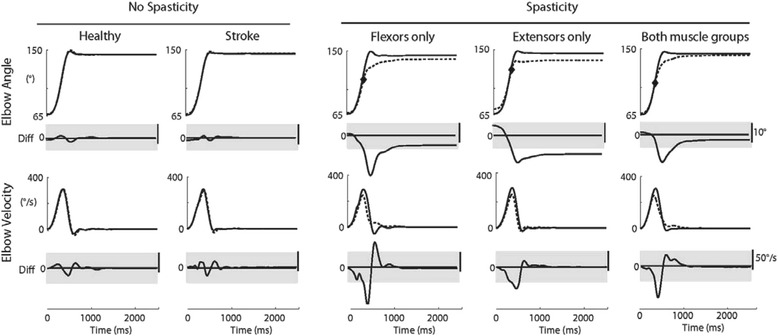
Exemplar participant traces from each subject group for extension movement (testing flexors). *Elbow angle* is plotted in the top traces for the affected (*stroke*) or non-dominant (*control*) limb represented by the *dotted lines* and unaffected or dominant limb represented by the *solid line*. Catch angle is indicated for the three participants with spasticity (*gray diamond*). Difference in elbow angle is plotted below (affected/non-dominant – unaffected/dominant). Elbow angular velocity is plotted in the bottom traces with velocity difference plotted below (affected/ non-dominant – unaffected/dominant)

Elbow flexor assessment during extension movement for exemplar participants are displayed in Fig. 2 for the fastest trials (600 ms duration). A Between-arm catch angle was observed in all spasticity subgroups, including the individual identified to have extensor only spasticity. Notably, the catch angle appeared later in the movement for this individual, with minimal change in trajectory prior to reaching the Final angle. Exemplar participants from the no spasticity group and the control group showed minimal between-arm differences.

Initial examination of participant response to passive stretch at five movement durations highlighted the ability to detect differences between groups was maximal during the fastest trials (600 ms). Slower movements did not substantively alter the results. Because the focus of the present study was on the development of a task to quantify resistance to movement in the context of clinical outcomes, data for the fastest trials only are presented in the remainder of the report.

### Control participant response to stretch

A normative model of response to passive stretch was developed from the healthy control group for all parameters except Between-arm catch angle (Table 2). No influence of age or handedness was found for any of these parameters, thus data for left and right arms were combined for healthy controls. Four of six parameters were influenced by the participant’s sex: Peak velocity (flexion), Final angle (flexion and extension), Between-arm peak velocity difference (flexion, and extension), and Between-arm creep difference (extension). Cut off values were calculated separately for males and females for these parameters. Three parameters could not be transformed to normal: Between-arm final angle difference (extension), Creep (flexion), and Between-arm creep difference (flexion). Impairment was identified when an individual from the patient population was not within cut-off values calculated from the control participants for each parameter.

### Individual participant response to stretch

**Fig. 3:**
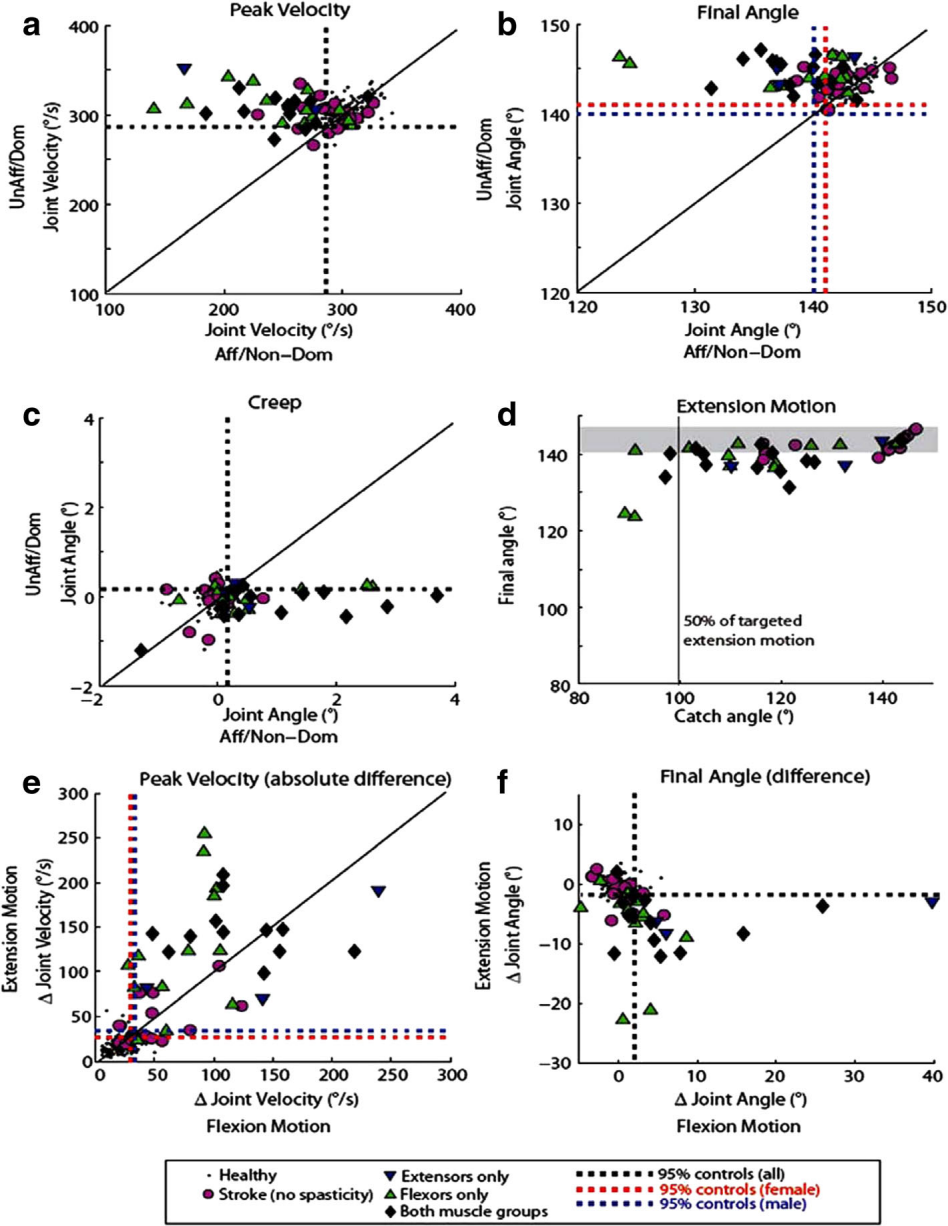
**a**-**c** Peak velocity, *final angle* and *creep parameters* compared across limbs for all participants for extension movement (*testing flexors*). *Dotted lines* represent cutoff values calculated from control behavior. Participants with stroke were considered impaired if they fell below the 5th percentile cutoff for peak velocity and final angle, and if they fell above the 95th percentile for creep. **d** Catch angle calculated for the extension movement compared to extension final angle (*affected arm*). *Gray line* represents the 50% of the targeted extension movement, *gray shaded* area represents the range of final angles for control participants. **e**-**f** Peak velocity and final angle difference (*affected/non-dominant - unaffected/dominant*) compared between flexion motion (*testing extensors*) and extension motion (*testing flexors*)

Virtually all individuals from the patient population were within the bounds of healthy controls when assessing the less affected arm for these parameters. Peak velocity of the more affected arm identified the majority of individuals in all spasticity subgroups, including the extensors only group, as impaired. Final angle and Creep parameters were not as effective at identifying impairment but separation of the spasticity groups and the no spasticity group was still observable. Peak velocity, Final angle, and Creep parameters for elbow flexor assessment during extension movement (i.e. test flexor muscles) are illustrated in Fig. 3a-c. Most of the individuals with no spasticity were within the bounds of healthy controls for these three parameters, although several of these individuals were also identified as impaired (Fig. 3a-c, Table 2).

Between-arm catch angle was observable for most participants in all spasticity subgroups and for 7 of 17 individuals in the no spasticity group (Fig. 3d). A catch can occur throughout the entire range of stretch though they were primarily observed in the latter half of the movement, as was the case for all individuals in the no spasticity group when a catch was present.

Between-arm peak velocity difference and Between-arm final angle difference were also effective at identifying the majority of individuals in all spasticity subgroups as impaired (Fig. 3e-f). However, between-arm differences were not always predictable using clinical scores. For example, one of three participants from the extensors only spasticity subgroup showed a large Between-arm peak velocity difference in both directions of movement (Fig. 3e, blue triangle in upper right quadrant). Similarly, an individual from the extensors only spasticity subgroup was near the bounds of healthy controls during flexion movement (testing extensors) but not for extension movement (Fig. 3e, blue triangle in lower left quadrant). Like all other parameters, some participants with no spasticity were identified as impaired.

**Fig. 4:**
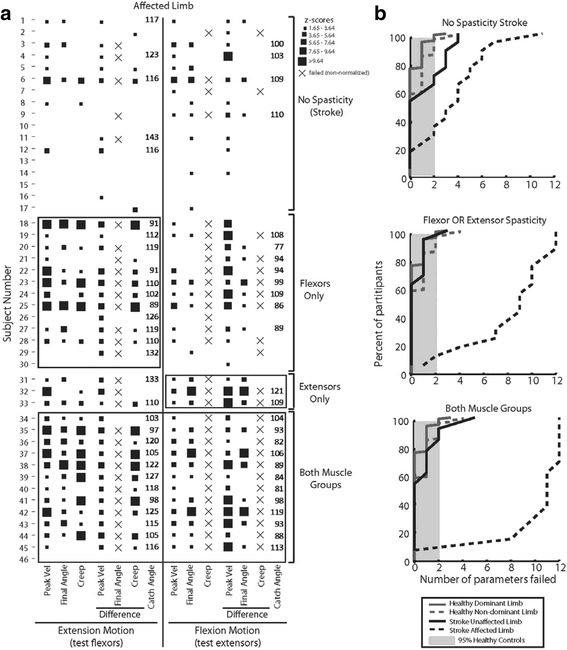
**a** Individual participant performance on each task parameter. The presence of a square indicates that the participant failed that parameter (behavior fell outside 95% of control performance). *Square size* indicates the z-score range, with *small squares* representing lower z-score values and *larger squares* representing higher z-score values (and worse normalized performance on the parameter). For those participants where it was detected, catch angle is reported. **b** Cumulative sum of the number of parameters failed for each participant group. *Dark gray* traces represent number of parameters failed by control participants, *black traces* represent number of parameters failed by stroke participants (affected/non-dominant arm, *dotted line*; unaffected/dominant arm, *solid line*). *Light gray* shaded area represents 95th percentile for controls. *Middle plot* combines Flexors only and Extensors only participant groups. Note that almost all participants identified with spasticity in both muscle groups (*bottom plot*) failed more parameters than 95% of all control participants

Actual failure rates and degree of failure varied between parameters (Fig. 4a). Between-arm peak velocity difference during flexion movements identified the greatest percentage of participants without spasticity as impaired (59%) (Fig. 4a, Table 2). Between-arm peak velocity difference also identified impairment when spasticity was present in the flexors only (85%), the extensors only (100%), or both muscle groups (92%) during extension and flexion movements. In the extensors only group, two of three participants showed impairment in all 7 parameters while one of three did not exhibit a Between-arm catch or Between-arm creep difference. One of three participants in the extensors only group failed all parameters during extension movement (testing flexors) though the z-scores were relatively low. Failure rate was highest across parameters when spasticity was present in both muscle groups, ranging from 77 to 92% during extension movements and 54 to 92% during flexion movements. Likewise, failure rate was high across parameters when spasticity was present in the extensors only, ranging from 67 to 100%, excluding Between-arm creep difference during extension (33%).

### Individual patterns of impairment

Participants displayed unique patterns of impairment, which in some cases did not reflect their classification using clinical measures and may be indicative of the increased sensitivity of the approach used in this study. For example, during extension movements, subject 30 did not fail any parameters despite a clinical presentation of flexor spasticity whereas subjects 6 and 33 each failed all extension parameters despite no clinical presentation of flexor spasticity. Similarly, subjects 23, 24, and 25 failed all parameters during flexion motion despite no clinical presentation of extensor spasticity. Cumulative sum of the number of parameters failed for each participant group are illustrated in Fig. 4b. These traces show that 85 to 90% of participants with clinically-identified spasticity failed more parameters than healthy controls.

### Intra-class correlation & clinical correlations

Intra-class correlations varied, depending on parameter, from 0.66 to 0.95 with the majority of correlations scoring above 0.8 (Table 2). The strongest correlations between two raters were for Peak velocity (ICC 0.90 for extension, ICC 0.87 for flexion), Between-arm peak velocity difference (ICC 0.95 for extension, 0.88 for flexion), and Between-arm final angle difference (ICC 0.91 for extension, 0.88 for flexion). This protocol was modestly correlated with the MAS and V2 and V3 of the MTS for most parameters. For extension and flexion movements, Between-arm creep difference was most strongly correlated with MAS (0.59 and 0.64 respectively). For the MTS, V2 was most strongly correlated to Between-arm creep difference (flexion; 0.58) and V3 was most strongly correlated to Creep (flexion; 0.57).

## Discussion

The objective of this study was to develop a tool to assess post-stroke spasticity in the upper limb. The KAPS protocol was designed to identify unique kinematic characteristics of resistance to passive movement in individuals with and without clinically-identified spasticity and provide a standardized methodology for assessment. Seven parameters were identified and tested to assess response to passive movement in both flexors and extensors: Peak velocity, Final angle, Creep, Between-arm peak velocity difference, Between-arm final angle, Between-arm creep, and Between-arm catch angle. These parameters identified impaired response to passive stretch not currently observable through clinical scores as resistance to passive movement is not exclusively dependent on hyperexcitability of the stretch reflex [32]. Clinical manifestation of spasticity was associated with high failure rate across all parameters in the extensors only (33–100% impaired), flexors only (38–85% impaired), and both muscle groups (54–92% impaired).

### Task development

The KAPS protocol employed a tightly controlled stretch of the elbow flexors and extensors through a defined range of 80° and assessed across 5 target durations of movement: 1500 ms, 1200 ms, 1000 ms, 800 ms, and 600 ms. This design was selected to mimic the standardization of the MAS and MTS while including faster and slower durations to better characterize the velocity-dependence of spasticity. Analyses revealed that the ability to detect differences between groups was optimized at the fastest duration of movement (600 ms). Beyond the objective of this study, future work may include analyses of all durations to assess within-group velocity-dependent differences. Final angle for healthy controls was within (or at the limits of) the 80° range, suggesting response to passive stretch is observable for this range. Importantly, the KAPS protocol identified most participants from the patient population as impaired including participants without clinically determined spasticity. This feature indicates that the components of the KAPS protocol has the sensitivity to detect subtle impairments in muscle tone that may still impact upper limb function, but which may be missed through routine clinical assessment.

Previous studies have advocated for assessing spasticity in conjunction with non-velocity dependent components of resistance to passive movement within a single measure. Lindberg and colleagues assessed the relationship between response to passive stretch and force contributions of muscle, passive elasticity, muscle viscosity, and inertial properties of the joint [33]. The complexity of these relationships makes clinical application difficult but improves our understanding of spasticity. The parameters identified through the KAPS protocol add clinical relevancy as they are easily replicable while highlighting the need for an all-encompassing assessment of spasticity as unique characteristics are being missed through traditional scales. Individuals identified as having spasticity in only the flexors or only the extensors through the MAS and MTS were often identified as impaired in both muscle groups through the KAPS protocol. Thus, the KAPS protocol supports the notion that spasticity impacts both muscles resisting gravity (i.e. elbow flexors) [34] and in antagonist muscle groups [35] and that the latter is more difficult to measure using the MAS and MTS. The results of this study suggest that current clinical scales may inappropriately classify spasticity in a single direction when resistance is present in both directions.

Interestingly, sex-differences were identified in 4 of the measures: Peak velocity (flexion), Final angle (flexion and extension), Between-arm peak velocity difference (flexion, and extension), and Between-arm creep difference (extension) and may be a result of sex-related differences in muscle properties. Body mass and size are typically different between sexes. In addition, joint position sense [36], joint displacement onset time [37], and muscle stiffness [38] have also been shown to be different for men and women. Thus, it is possible that in our patient cohort, the sex-differences in some measures of the KAPS reflect differences that are inherent in muscle properties and limb kinematics.

### Quantitative characteristics of spasticity

Peak velocity was the most effective parameter at identifying impairment in all spasticity subgroups. In isolation, a lower Peak velocity indicates resistance to passive movement in the first half of the movement. Between-arm peak velocity differences, using the less affected side as a reference, identified the greatest percentage of participants from the patient population without spasticity as impaired (59%). These two parameters also showed the strongest intra-class correlations indicating high consistency between raters. Taken together with the high patient failure rate, these two parameters are suited to differentiate the patient population from healthy controls.

An ‘earlier’ Final angle (lower movement extent) and larger Between-arm final angle difference is indicative of tissue shortening, or contracture [39]. After the stretch, the robot generates a torque proportional to the difference between the Final angle and the specified end angle (0.26 Nm/deg), attempting to drive the arm to maximal range. This allows assessment of the extent of resistance to movement after the initial stretch reflex when resistance persists if the stretch is maintained [40]. There was a clear separation between groups when assessing these parameters suggesting that a reduced passive range is associated with clinical spasticity. Though this study did not include a clinical measure of contracture, these results suggest that tissue shortening has occurred and is more likely in the presence of spasticity [15, 32].

Spasticity has historically been assessed in terms of joint position at the occurrence of resistance (catch) and degree of resistance through the remainder of the movement (release). Between-arm catch angle was defined in this study through a velocity difference threshold for an extensive control population. Between-arm catch angle was observable for individuals in all spasticity subgroups and the no spasticity group between 24° after movement onset and 64°. This kinematic information may support the view that there is an inability to relax muscles at a specific point within the biomechanical range for an individual [19, 41]. All participants that exhibited a catch despite no clinical spasticity did so within the last 35° of the movement. Creep and Between-arm creep difference parameters further separated those with clinical spasticity from those without. These results suggest the classic ‘catch and release’ associated with spasticity is captured through the KAPS protocol.

Importantly, analysis for consistency between raters was conducted on individuals ranging from none-to-moderate levels of spasticity as quantified by clinical measures. This indicates sensitivity of the KAPS to characterize resistance to movement across a range of severities. However, it must be acknowledged that the cohort lacks individuals with high levels of clinically-measured spasticity. Thus, interpretation of the study results must be taken in the context of level of severity. Future studies must determine the applicability of the KAPS in individuals with higher severity.

In correlating KAPS outcomes to MTS and MAS scores, the analyses revealed moderate-to-strong correlations in all measures, except between-limb differences in peak velocity in both flexion and extension. In addition, the correlations were low at slower movement speeds of the MTS (V2 and V3). Intuitively, it is reasonable to expect the MAS and MTS (single limb measures) should not be associated with between-limb (dual limb measures). It is also intuitive that the between-limb differences in peak velocity at the fast movement of the KAPS should not be associated with slower movements of the MTS. Future studies will examine interrelationships between clinical and kinematic measures across a range of speeds.

### Limitations

In spite of the strengths of the study, there are limitations that must be acknowledged. The KAPS protocol is one that was developed to characterize velocity-dependent and velocity-independent features of resistance to passive movement. However, the results of the current study only address those features associated with velocity-dependent movement. Future studies will examine characteristics of each phenomenon. In addition, the KAPS was developed as a tool to better inform clinical assessment of spasticity. While costs associated with the device, time, and specificity of assessment to specific muscles may limit broader clinical utility, the findings do advance techniques and measures that may be clinically relevant.

Though excellent consistency was observed across 3 trials at a given speed, the KAPS protocol may benefit from additional trials. Future iterations of the task may also benefit from randomization of the arm tested first and the order of stretch durations. A further limitation of this protocol is the variable starting angle determined by the passive range of each individual participant. Further evaluation of the KAPS protocol may benefit from determining the extent to which variable starting positions influence each parameter.

## Conclusions/clinical implications

Traditional assessments of spasticity have relatively coarse scales that do not capture all characteristics of the impairment and may misclassify individuals. The KAPS protocol identified three participants that failed all parameters in both directions, and yet were classified as having spasticity in only a single muscle group. Fourteen participants without spasticity failed at least 1 parameter. The KAPS protocol identified unique characteristics of clinical spasticity and provided a standardized methodology for assessment. Future analyses will focus on assessing the velocity-sensitive nature of the parameters proposed in this study. Further work is needed to identify sensitivity to change in response to anti-spastic medications and treatments. The protocol presented here may provide a useful quantification of spasticity that may guide future work into the influence of resistance to passive movement on function.

## Abbreviations

Arm score: CMS Aarm, Chedoke-McMaster Stroke Assessment
EMG: Electromyography
ICC: Intra-class correlation
KAPS: Kinematic Assessment of Passive Stretch
MAS: Modified Ashworth Scale
MTS: Modified Tardieu Scale
